# Analysing the Operative Experience of Paediatric Surgical Trainees in Sub-Saharan Africa Using a Web-Based Logbook

**DOI:** 10.1007/s00268-020-05892-6

**Published:** 2020-12-08

**Authors:** Ciaran Mooney, Sean Tierney, Eric O’Flynn, Miliard Derbew, Eric Borgstein

**Affiliations:** 1 Faculty of Medicine, Health and Life Sciences, Queen’s University Belfast, Belfast, United Kingdom; 2 Department of Surgical Affairs, Royal College of Surgeons in Ireland, Dublin, Ireland; 3 Institute of Global Surgery, Royal College of Surgeons in Ireland, Dublin, Ireland; 4 School of Medicine, Addis Ababa University, Addis Ababa, Ethiopia; 5 Department of Surgery, University of Malawi, Blantyre, Malawi

## Abstract

**Background:**

The expansion of local training programmes is crucial to address the shortages of specialist paediatric surgeons across Sub-Saharan Africa. This study assesses whether the current training programme for paediatric surgery at the College of Surgeons of East, Central and Southern Africa (COSECSA) is exposing trainees to adequate numbers and types of surgical procedures, as defined by local and international guidelines.

**Methods:**

Using data from the COSECSA web-based logbook, we retrospectively analysed numbers and types of operations carried out by paediatric surgical trainees at each stage of training between 2015 and 2019, comparing results with indicative case numbers from regional (COSECSA) and international (Joint Commission on Surgical Training) guidelines.

**Results:**

A total of 7,616 paediatric surgical operations were recorded by 15 trainees, at different stages of training, working across five countries in Sub-Saharan Africa. Each trainee recorded a median number of 456 operations (range 56–1111), with operative experience increasing between the first and final year of training. The most commonly recorded operation was inguinal hernia (*n* = 1051, 13.8%). Trainees performed the majority (*n* = 5607, 73.6%) of operations recorded in the eLogbook themselves, assisting in the remainder. Trainees exceeded both local and international recommended case numbers for general surgical procedures, with little exposure to sub-specialities.

**Conclusions:**

Trainees obtain a wide experience in common and general paediatric surgical procedures, the number of which increases during training. Post-certification may be required for those who wish to sub-specialise. The data from the logbook are useful in identifying individuals who may require additional experience and centres which should be offering increased levels of supervised surgical exposure.

## Introduction

Children make up half of the population of Sub-Saharan Africa [1]. It has been estimated that as many as 85% of them will develop a surgically treatable condition by the time they turn fifteen [2]. Despite this, paediatric surgical services across much of the continent remain heavily underdeveloped, with a severe shortage of specialist surgeons [3–6].

Expansion of national surgical training programmes is crucial in addressing workforce shortages and strengthening healthcare systems in low- and middle-income countries (LMICs) [7, 8]. The data recorded in surgical logbooks are considered as a major indicator of the quality of these training programmes [9], enabling continuous trainee assessment by determining operative exposure and analysing training post suitability through levels of supervision [10]. Integration of electronic logbooks (eLogbooks) into surgical training curricula has enabled the production of large standardised and centralised datasets [11]. Whilst there have been a number of publications that have studied the use of eLogbooks for surgical training in high-income countries, few have studied their use in Sub-Saharan Africa or in other LMICs, and none have specifically looked at eLogbooks in the context of paediatric surgical trainees [11–21].

The College of Surgeons of East, Central and Southern Africa (COSECSA) delivers postgraduate surgical education and training in 14 member countries in the Sub-Saharan region. This study uses the COSECSA eLogbook to analyse the operative experience of trainees enrolled on the Fellowship Training and Examination in Paediatric surgery (FCS Paedsurgery (ECSA)), helping to determine whether the current training programme for paediatric surgery is exposing trainees to adequate numbers and types of surgical procedures, under sufficient levels of supervision. Identification of gaps in the current training programme will provide actionable insight into how paediatric surgical training can be improved in the region.

## Materials and methods

### Study population

Since 2015, all new COSECSA trainees have been required to keep a record of their operative experience as a mandatory aspect of their training programme, using a password protected web-based logbook (https://logbook.cosecsa.org). The eLogbook replaced the old paper-based logbooks and was developed in partnership with the Royal College of Surgeons in Ireland (RCSI).

As of midnight, on 31/12/2019, there were a total of 211, 509 operations recorded in the COSECSA eLogbook.

## Selection and data collection

Operations recorded in the eLogbook between 1^st^ January 2015 and midnight on 31^st^ December 2019 by trainees enrolled on the FCS Paedsurgery (ECSA) programme were eligible for analysis. To be included in the present study, the trainee must have completed the full year of training in question and recorded at least 50 operations per year. Procedures carried out by trainees exempt from compulsory logbook use due to prior enrollment in a COSECSA programme when the eLogbook was introduced were excluded from analysis. Additionally, procedures carried out by COSECSA trainees who were based in a Pan African Academy of Christian Surgeons (PAACS) hospital when the procedure was carried out were also excluded. PAACS hospitals already use an eLogbook which has been deemed adequate to produce the assessment data required by COSECSA.

Three of the trainees recorded (546) operations they had performed outside of Sub-Saharan Africa during exchange programmes. Whilst we included these operations in determining trainee experience, the entries were excluded from analysis of hospital, patient and mortality demographics as they do not reflect regional surgical burden or capacity.

## Data analysis

Table 1 lists the fields which trainees must fill in when entering the data in the COSECSA eLogbook. Additional information on the gender, the training programme and the programme year of the trainee at the time of the operation was obtained from the COSECSA central database and matched with the eLogbook data.

The eLogbook automatically sorts each operation into one of 11 ‘bundles’, based on the type of surgery carried out. Patient age was categorised using the World Health Organization (WHO) position paper on Paediatric Age Categories for Essential Medicines Lists for Children, creating five distinct age groups [22]. Patient ID was removed prior to analysis.

Retrospective analysis of the eLogbook data was subsequently performed, with operative experience of the trainees compared with both COSECSA guideline numbers and the Joint Committee on Surgical Training (JCST) indicative trajectory index case numbers for those at the end of Specialty Training 5 (ST5), three years into paediatric surgical training in the UK. [23]

## Statistics

Data were extracted from the eLogbook onto Microsoft Excel [24] and converted to IBM SPSS Statistics version 25.0 [25] for analysis. Descriptive analysis of the data was reported as frequencies, percentages, medians and interquartile ranges.

## Results

From 8335 eligible eLogbook entries, 7616 (91.4%) were included in the final analysis. 175 operations carried out on adult patients by trainees during their FCS Paedsurgery (ECSA) programme were excluded, as well as a further 185 operations which were recorded by a trainee during an extra year of training.

### Demographics

Entries from 15 paediatric surgical trainees (eight females, seven males) at different stages of training were included in the analysis, with each trainee recording a median 456 (range 56–1111) operations in the eLogbook. The trainees worked across 14 centres in five Sub-Saharan countries: Kenya, Malawi, Uganda, Zambia and Zimbabwe. Most operations (93.1%, *n* = 6582) were carried out in public hospitals across the region (Table 2). A small number of operations (0.2%, *n* = 11) were performed at two hospitals in Uganda and Zambia which were unaccredited by COSECSA. Of the twelve COSECSA accredited hospitals, nine were in large cities, where 93.3% (*n* = 6593) of the operations were carried out. A total of 466 (6.6%) operations were carried out in three hospitals in smaller towns. As trainees enter the hospital name in the eLogbook from a drop-down list of accredited hospitals, it is unclear where exactly the unaccredited hospitals were situated in Uganda and Zambia.

**Table 1:**
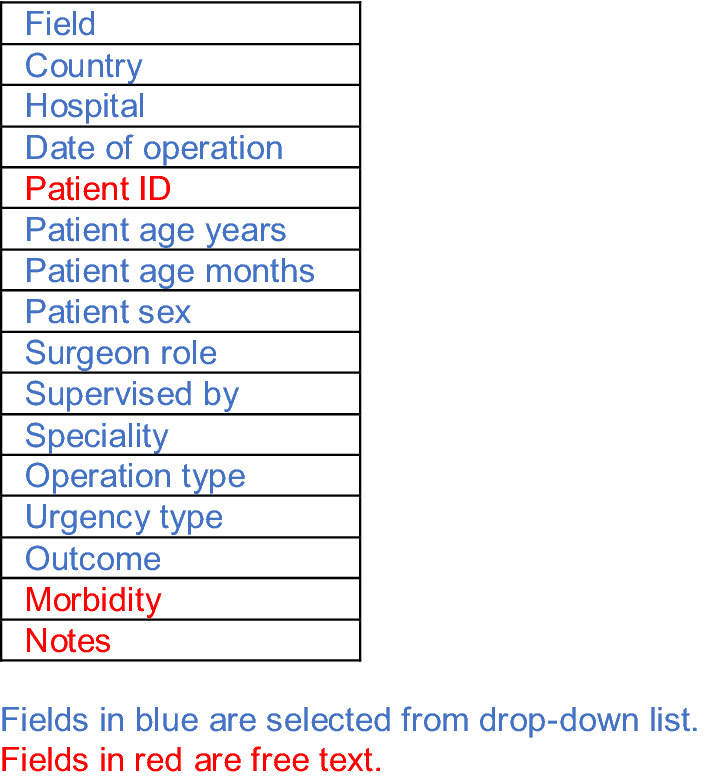
COSECSA eLogbook fields

**Table 2:** Number of operations by hospital type

Country	Public hospital (%)	Faith-based/ NGO hospital (%)	Unaccredited hospital (%)	Total (%)
Kenya	428 (49.0)	445 (51.0)	0 (0.0)	873 (100.0)
Malawi	1745 (100.0)	0 (0.0)	0 (0.0)	1745 (100.0)
Uganda	1576 (99.7)	0 (0.0)	4 (0.3)	1580 (100.0)
Zambia	1408 (97.3)	32 (2.2)	7 (0.5)	1447 (100.0)
Zimbabwe	1425 (100.0)	0 (0.0)	0 (0.0)	1425 (100.0)
Total across all countries	6582 (93.1)	477 (6.7)	11 (0.2)	7070 (100.0)

**Fig.1:**
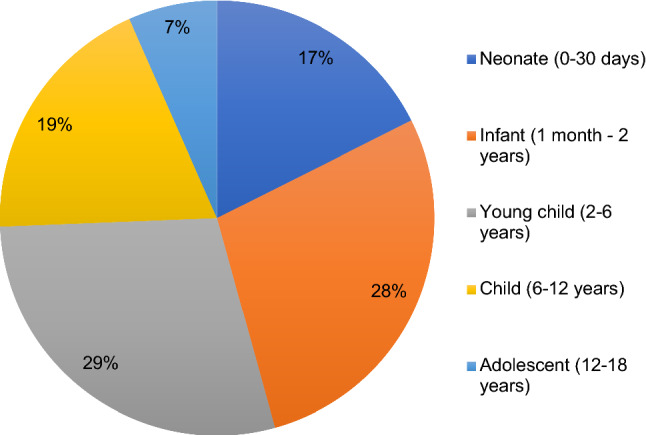
Patient age categories

‘Young children’ were the most common patient age group (Fig. 1). The median age of the patients operated on across Sub-Saharan Africa was 2.00 (IQR = 0.50–6.00) years old. The majority (*n* = 4661, 65.9%) of paediatric patients operated in all five countries were male.

Most (*n* = 4900, 69.3%) operations performed within the Sub-Saharan Africa region were carried out electively, with 2170 (30.7%) emergency procedures recorded. A previous study determined that an emergency to elective (Ee) ratio, which represents the number of emergency surgeries performed for every 100 elective surgeries, is a good indicator of access to available surgical care and perioperative mortality risk [26]. The Ee ratio for these data is 44.3.

Analysis of the operative outcomes in this study determined a mortality rate of 3.8% (*n* = 269). A further 160 operations (2.3%) resulted in deterioration of the patient’s condition, and a small number (*n* = 16, 0.2%) of patients were discharged against medical advice.

### Trainee operative experience

**Fig. 2:**
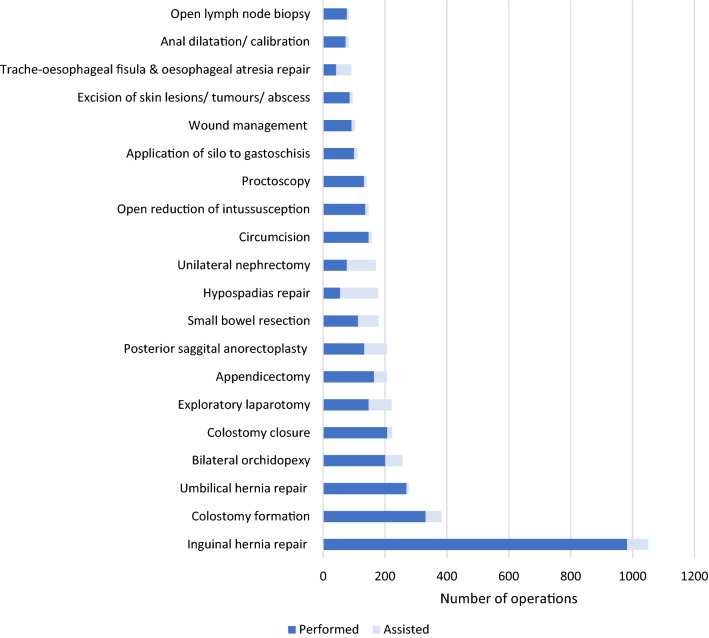
Proportion of the 20 most commonly recorded operations performed/assisted over the data period

**Fig. 3:**
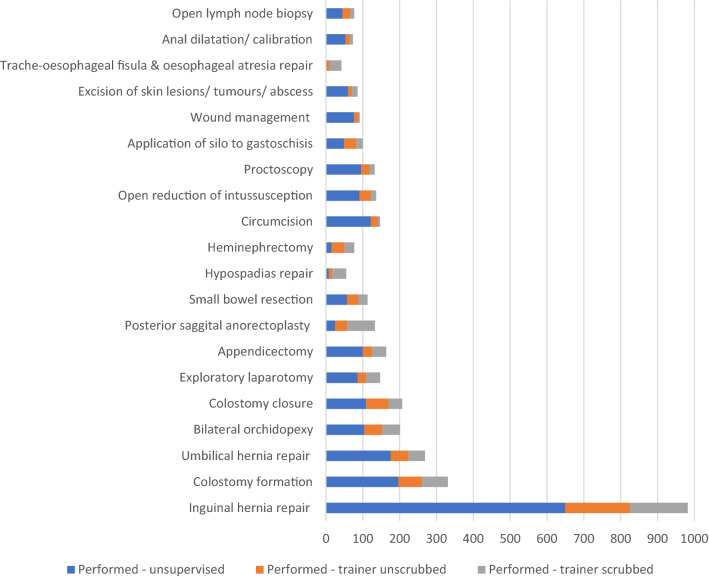
Breakdown of the 20 most commonly recorded operations which were ‘performed’ by trainees

‘*Inguinal hernia repair’* was the most commonly recorded operation, accounting for 1051 (13.8%) of the entries in the eLogbook (982 performed, 69 assisted), followed by ‘*colostomy formation*’ and ‘*umbilical hernia repair’* (Fig. 2). Trainees performed the majority (*n* = 5607, 73.6%) of operations recorded in the eLogbook themselves, assisting in the remainder. Those *performed* are classified further in Fig. 3, according to whether their supervisor was present and ‘scrubbed in’. The most commonly performed operations were similar between different centres and countries, except for two hospitals, both in Zambia, where only plastic surgery operations were recorded as having taken place.

**Fig. 4:**
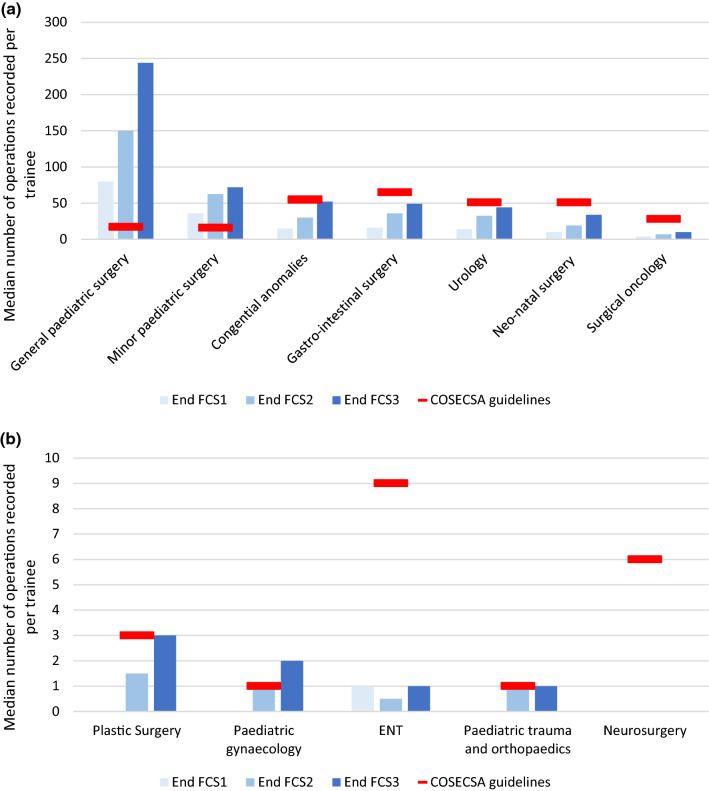
**a** and **b**: Median number of operations carried out in each bundle per trainee per year of training, compared with the COSECSA guideline number for the FCS Paedsurgery programme

As shown in Fig. 4a, b, the median number of operations recorded per trainee increased between year one and three of the programme in all bundles, except neurosurgery and ENT.

Using data from the nine trainees who completed all three years of training during the study period, Table 3 shows that the average trainee recorded an acceptable proportion (75% or more) of the COSECSA guideline case numbers for 7.5 out of 12 bundles. All trainees exceeded the guideline numbers by at least 500% for general paediatric surgery and 200% for minor paediatric surgery. Completion rates were much smaller in sub-specialist areas such as paediatric neurosurgery and surgical oncology.

**Table 3:**
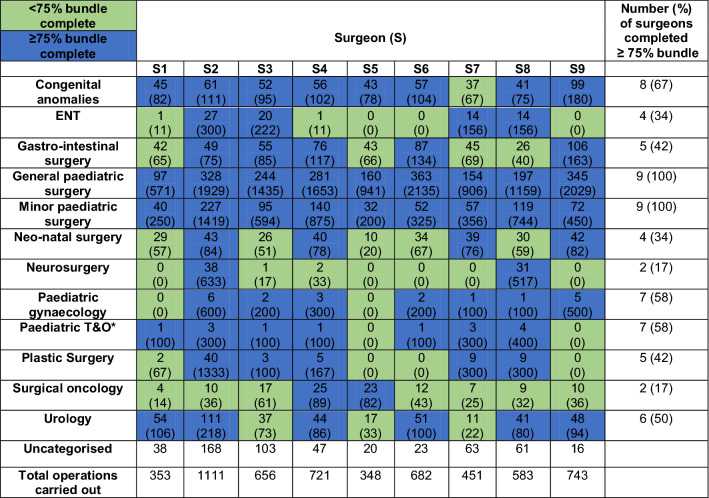
Number of operations in each bundle carried out by surgeons who completed FCS1-3 (% of COSECSA guideline number completed) *T&O = trauma and orthopaedics

**Fig.5:**
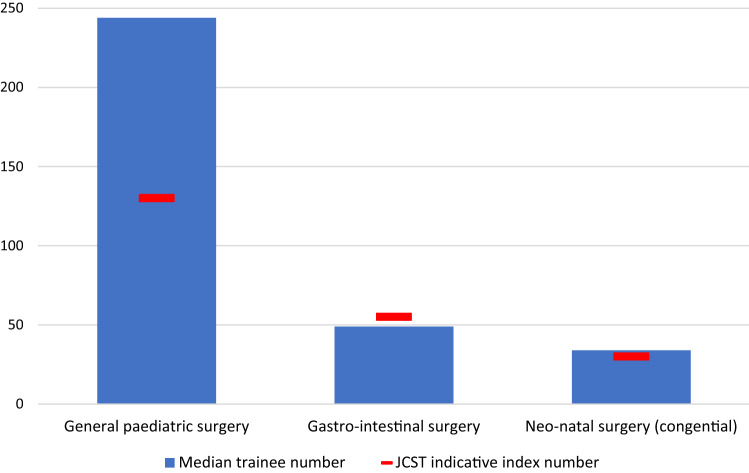
Median number of operations in each bundle carried out by COSECSA trainees (who have completed FCS1-3) compared with JCST indicative index number for ST5 paediatric surgical trainees (those who have completed 3 years of UK training)

Figure 5 shows the median number of operations carried out by COSECSA trainees (who have completed FCS1-3) and compares them with the JCST indicative index number for ST5 (year three) paediatric surgical trainees in the UK for similar sub-speciality groupings (where available). Similar to their performance in relation to the COSECSA guideline numbers, trainees greatly exceeded the number of general paediatric operations required, whilst underperforming in more specialist areas.

Trainees recorded a significant number of operations which were not categorised by the eLogbook.

## Discussion

This study provides a clear view of the level of operative experience obtained by trainees on a specialist paediatric surgical training programme in Sub-Saharan Africa. Paediatric surgical trainees are getting sustained and in-depth exposure to common paediatric surgical conditions. The number of more complex specialised procedures is smaller and indicates that it will be necessary to closely monitor exposure of individual trainees in this area. In addition, those who intend to practice in sub-specialist areas may require post-certification fellowship before commencing independent practice in sub-specialist areas of practice.

Whilst the sample size is relatively small, reflective of the small numbers training in paediatric surgery in the region, our analysis shows high variation in the level of operative experience that individual trainees gain. This may be consistent with research from high-income countries where underreporting of operative exposure by trainees is common [11, 27]. Furthermore, trainees may not be consistently recording operations throughout the programme and instead enter all records in the final year of training, when the logbook is due to be assessed. Periodic assessment of the logbook throughout the three-year programme may encourage more consistent use. It is however worth noting that trainees are also exposed to paediatric surgery in their basic membership training, where children account for 27% of the patient cohort [28].

As expected from a high-quality surgical training programme, analyses found that in the majority of bundles, the median number of operations carried out increased between years one and three of the programme. Most operations were also performed by trainees (as opposed to assisted in), making it clear that surgeons receive adequate opportunity to carry out operations independently before qualifying.

Our study shows that by both local and international standards, paediatric surgical trainees in the COSECSA region are gaining ample experience carrying out common general surgical procedures such as inguinal hernia repair and colostomy formation. However, in narrow sub-specialist areas where there is simply inadequate volume for trainees to become competent, post-training fellowships may be required to upskill those who intend to practise in those areas. The logbook data may be of particular value in identifying COSECSA surgical training hospitals that can offer appropriate exposure in sub-specialities, for example plastic surgery, during training.

Whilst the majority of operations in our sample were carried out on an elective basis, the Ee ratio is high (the ideal ratio is likely close to 5.5), albeit considerably lower than that reported by Prin et al. for all countries in Sub-Saharan Africa (62.6 (IQR 17.8–111.0)) [26]. It is however possible that the larger urban centres where paediatric surgical training is centralised will see a higher proportion of elective and/or more complex cases in contrast to regional and district hospitals where the proportion of emergency surgery may be higher [29].

The mortality rate of 3.8% recorded in the eLogbook exceeds that which has been documented in high-income countries (0.7%) [30]. However, there are particularly important limitations to the accuracy of these data. It is unknown how consistently trainees are made aware of those patients who die in hospital in the time following surgery, and furthermore, how consistently trainees then revisit their logbook records to reflect changed outcomes. It can also be assumed that the death of a patient following discharge is not recorded. Trainee logbooks are not assessed on mortality or morbidity.

Most of the children operated on by trainees in the region were males, with fewer operations carried out on female patients in all five countries. Research indicates that male infants are at increased risk of birth defects than female infants [31], and particularly relevant to this cohort, inguinal hernias affect up to nine times more males than females [32]. Further research into gender differences between paediatric surgical patients in the region, and indeed the reasons behind these differences, is required. Furthermore, very few surgical procedures were recorded on adolescents. One can speculate that children of this age are more commonly operated on by other surgeons or other cadres of healthcare worker.

The majority of the operations in our dataset were carried out at hospitals situated in large cities across the region. While it is unclear where the patients in this dataset travelled from, it is clear that the majority of the population of the region live in rural areas far from specialist paediatric surgical services and would need to travel long distances to access such services [33, 34]. This likely contributes to the late presentation and advanced pathology commonly seen across the continent, as well as the high mortality rate. Further efforts should be made to explore how access might be improved for those living in rural areas who require appropriate specialist care and might include incentives for early career surgeons to take up positions in more rural hospitals and investment to ensure that these facilities are appropriately resourced for specialised surgery.

## Limitations

Several limitations of this study should be noted. The data presented contain information from a small number of trainees in a small number of centres, over a relatively short period of time. As has been found in other studies, trainees may not record all the procedures they perform in their logbook, resulting in underestimation of their total operative experience. Additionally, trainees often carry out a large number of paediatric surgical operations before joining the FCS Paedsurgery (ECSA) programme. Therefore, the resulting data may not be entirely reflective of the true status of paediatric surgical training across the COSECSA region, and indeed Sub-Saharan Africa.

## Conclusions

This study has reported for the first time a multi-centre, multi-country overview of the operative experience of trainees within a structured supervised specialist programme of training in paediatric surgery in Sub-Saharan Africa. Trainee eLogbooks show that all obtain a wide experience in common general paediatric surgical procedures, with experience increasing throughout the programme. Logbook data can be used to make trainees aware of areas where more operative exposure is required, to indicate training centres which might offer niche exposure in particular areas and to help training programme directors ensure that all training centres are providing appropriately supervised operative experience for trainees assigned to them.
